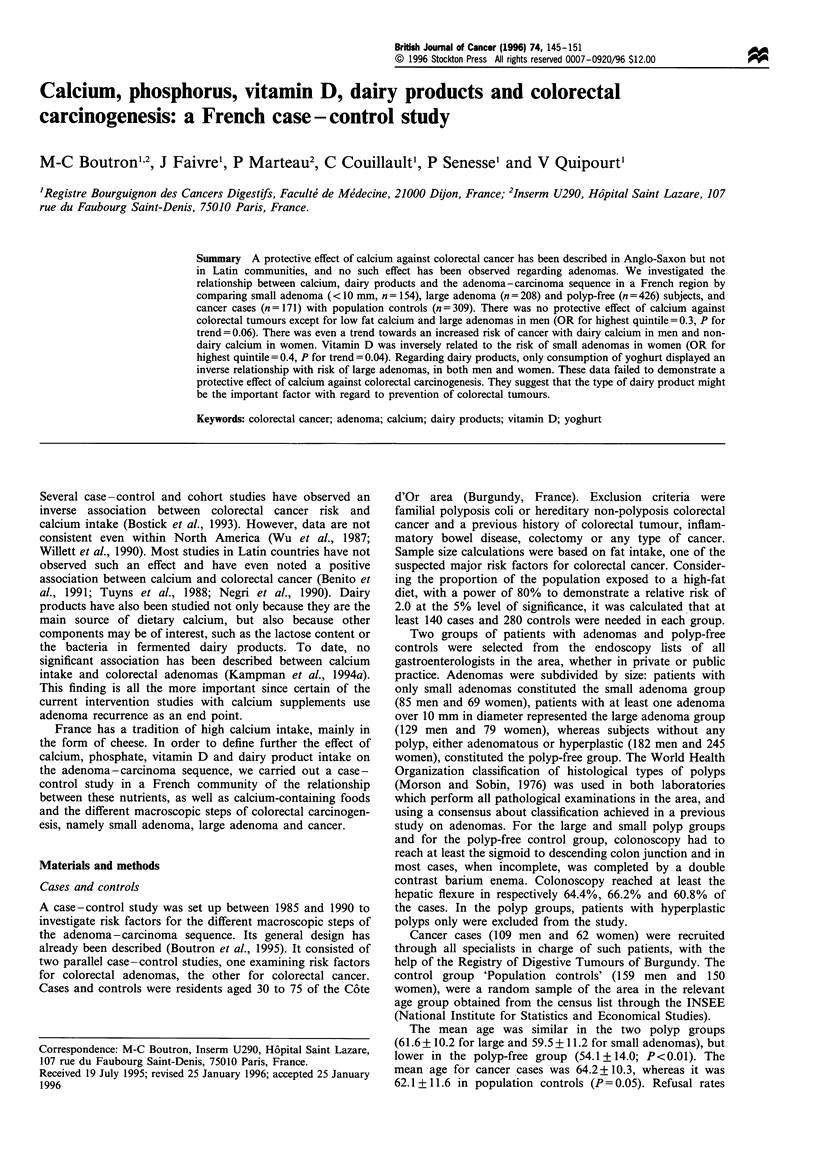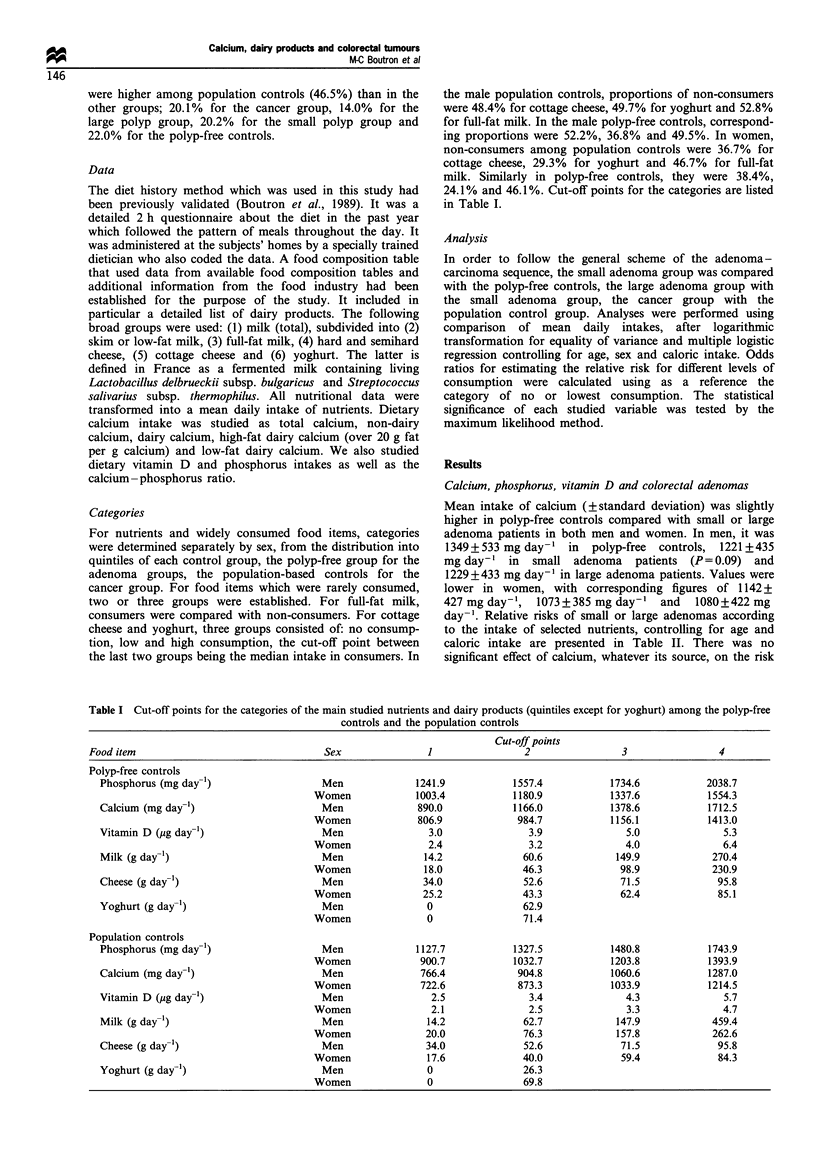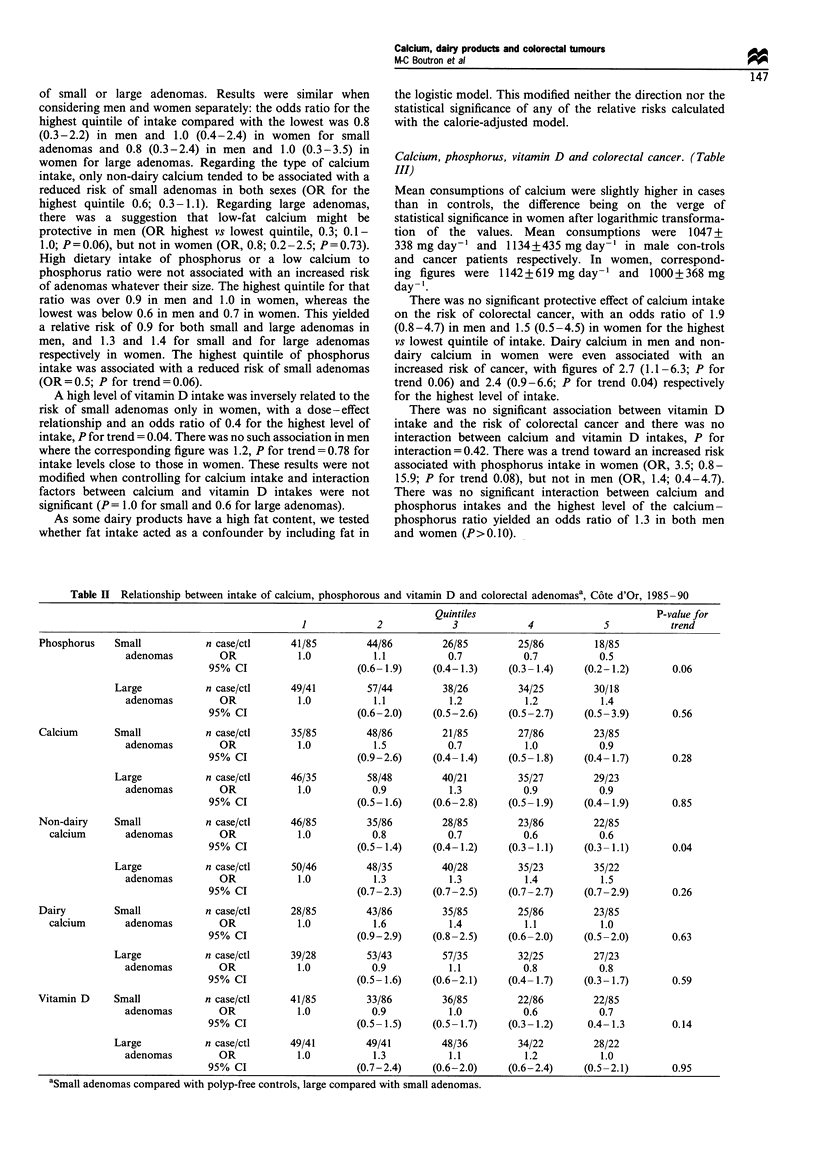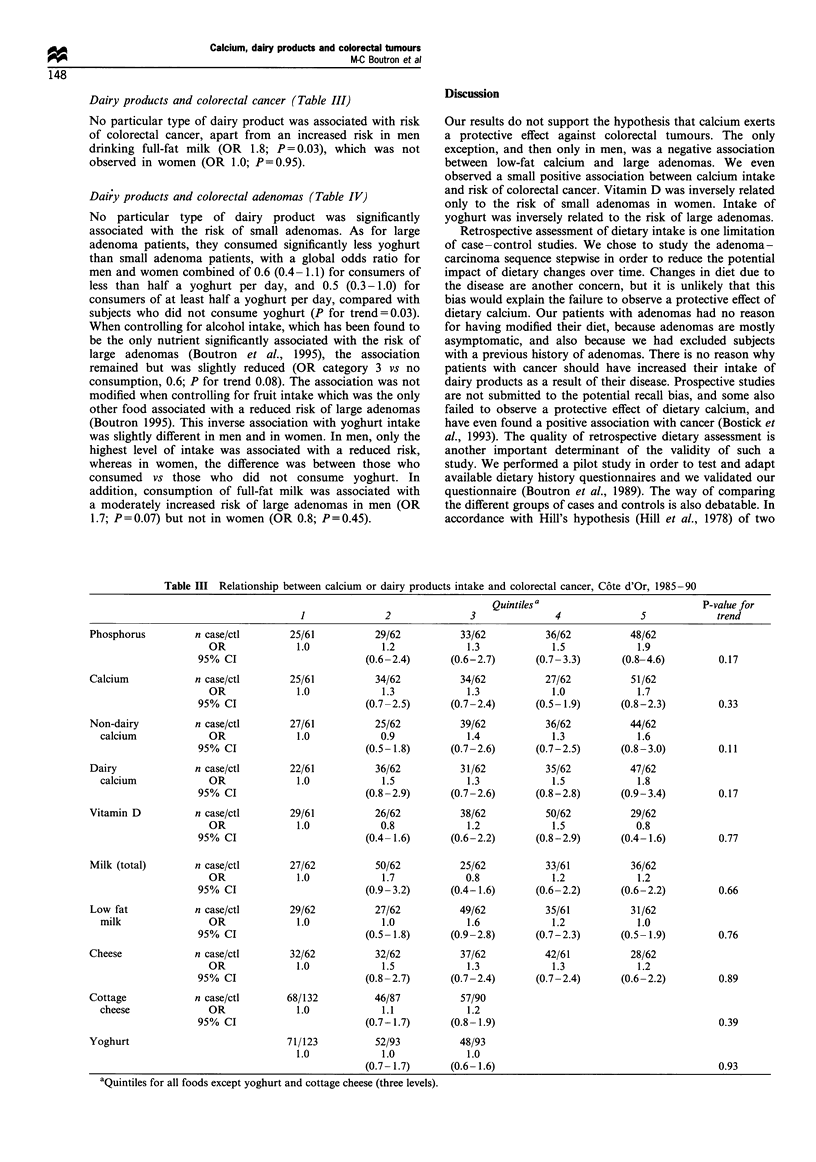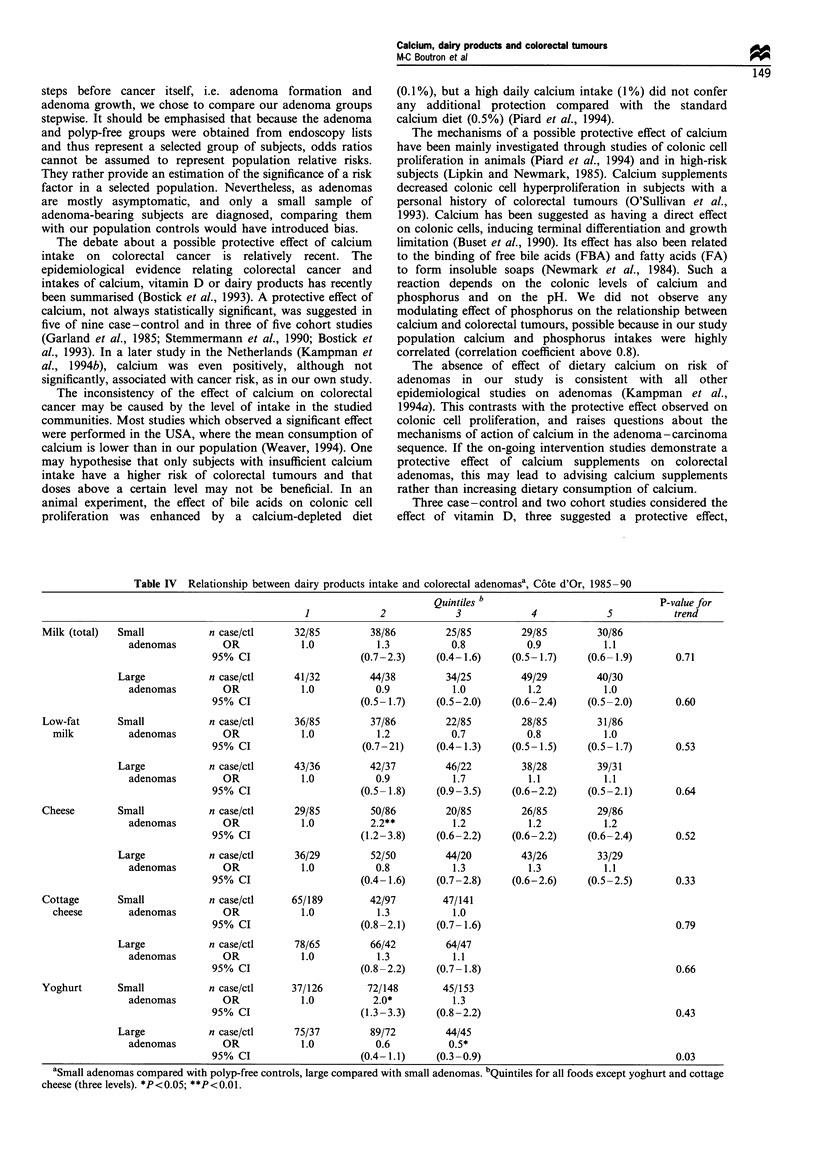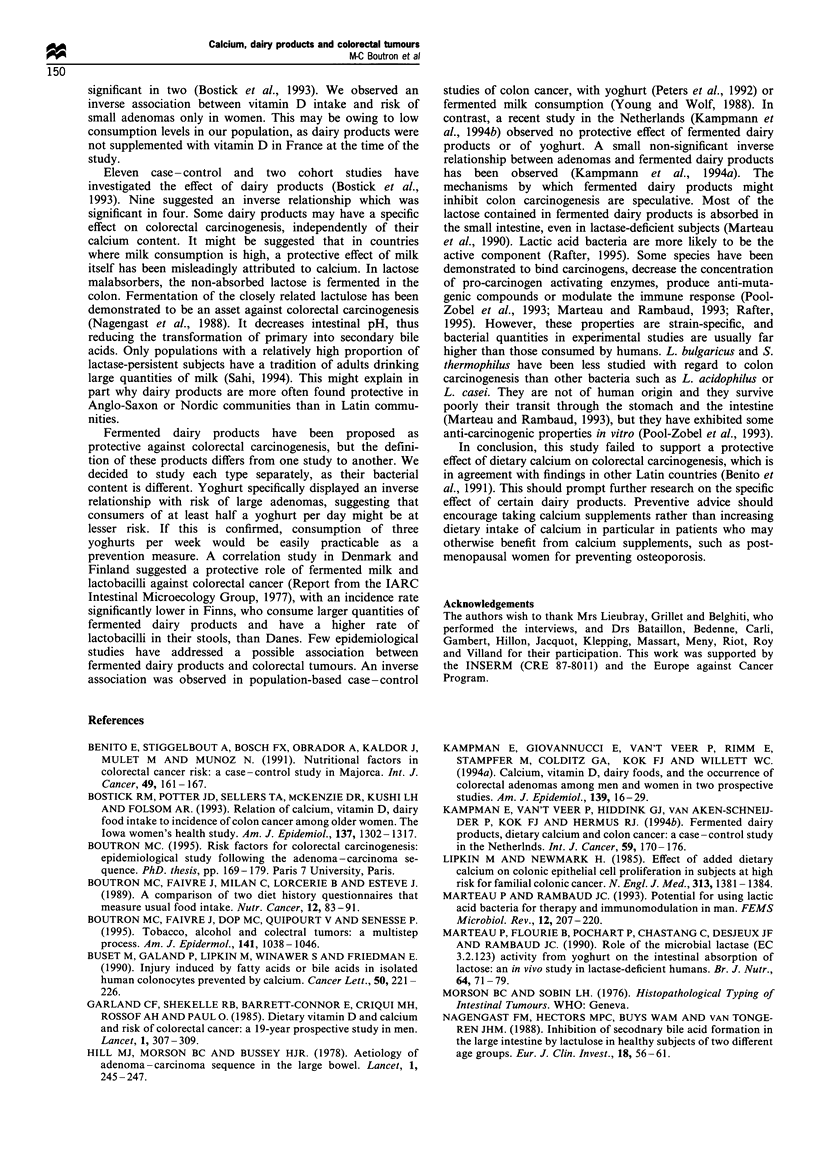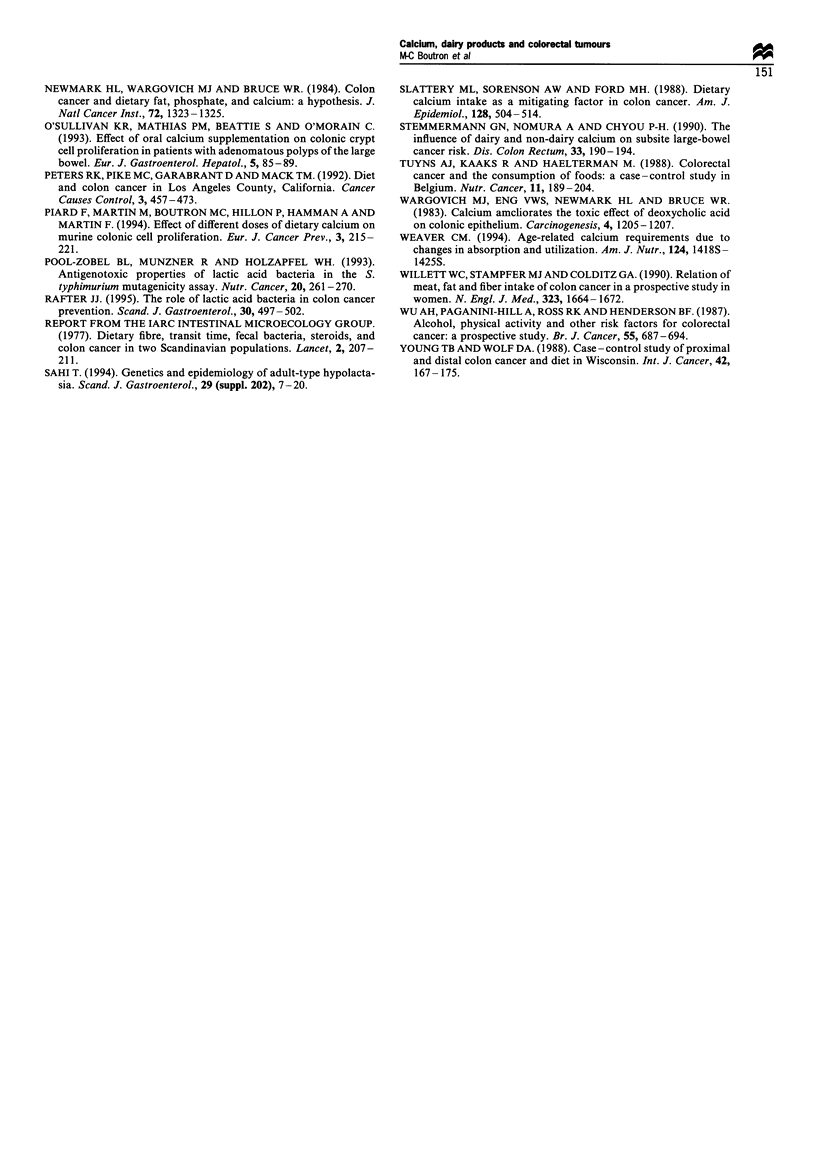# Calcium, phosphorus, vitamin D, dairy products and colorectal carcinogenesis: a French case--control study.

**DOI:** 10.1038/bjc.1996.330

**Published:** 1996-07

**Authors:** M. C. Boutron, J. Faivre, P. Marteau, C. Couillault, P. Senesse, V. Quipourt

**Affiliations:** Registre Bourguignon des Cancers Digestifs, Faculté de Médecine, Dijon, France.

## Abstract

A protective effect of calcium against colorectal cancer has been described in Anglo-Saxon but not in Latin communities, and no such effect has been observed regarding adenomas. We investigated the relationship between calcium, dairy products and the adenoma-carcinoma sequence in a French region by comparing small adenoma ( < 10 mm, n = 154), large adenoma (n = 208) and polyp-free (n = 426) subjects, and cancer cases (n = 171) with population controls (n = 309). There was no protective effect of calcium against colorectal tumours except for low fat calcium and large adenomas in men (OR for highest quintile = 0.3, P for trend = 0.06). There was even a trend towards an increased risk of cancer with dairy calcium in men and non-dairy calcium in women. Vitamin D was inversely related to the risk of small adenomas in women (OR for highest quintile = 0.4, P for trend = 0.04). Regarding dairy products, only consumption of yoghurt displayed an inverse relationship with risk of large adenomas, in both men and women. These data failed to demonstrate a protective effect of calcium against colorectal carcinogenesis. They suggest that the type of dairy product might be the important factor with regard to prevention of colorectal tumours.


British Journal of Cancer (1996) 74, 145-151
© 1996 Stockton Press All rights reserved 0007-0920/96 $12.00

### Calcium, phosphorus, vitamin D, dairy products and colorectal carcinogenesis: a French case-control study

M-C Boutron[1,2], J Faivre[1], P Marteau[2], C Couillault[1], P Senesse[1] and V Quipourt[1]

[1]*Registre Bourguignon des Cancers Digestifs, Faculté de Médecine, 21000 Dijon, France;* [2]*Inserm U290, Hôpital Saint Lazare, 107 rue du Faubourg Saint-Denis, 75010 Paris, France.*

**Summary** A protective effect of calcium against colorectal cancer has been described in Anglo-Saxon but not in Latin communities, and no such effect has been observed regarding adenomas. We investigated the relationship between calcium, dairy products and the adenoma-carcinoma sequence in a French region by comparing small adenoma (<10 mm, $n=154$), large adenoma ($n=208$) and polyp-free ($n=426$) subjects, and cancer cases ($n=171$) with population controls ($n=309$). There was no protective effect of calcium against colorectal tumours except for low fat calcium and large adenomas in men (OR for highest quintile = 0.3, $P$ for trend = 0.06). There was even a trend towards an increased risk of cancer with dairy calcium in men and non-dairy calcium in women. Vitamin D was inversely related to the risk of small adenomas in women (OR for highest quintile = 0.4, $P$ for trend = 0.04). Regarding dairy products, only consumption of yoghurt displayed an inverse relationship with risk of large adenomas, in both men and women. These data failed to demonstrate a protective effect of calcium against colorectal carcinogenesis. They suggest that the type of dairy product might be the important factor with regard to prevention of colorectal tumours.

**Keywords:** colorectal cancer; adenoma; calcium; dairy products; vitamin D; yoghurt

Several case-control and cohort studies have observed an inverse association between colorectal cancer risk and calcium intake (Bostick *et al.*, 1993). However, data are not consistent even within North America (Wu *et al.*, 1987; Willett *et al.*, 1990). Most studies in Latin countries have not observed such an effect and have even noted a positive association between calcium and colorectal cancer (Benito *et al.*, 1991; Tuyns *et al.*, 1988; Negri *et al.*, 1990). Dairy products have also been studied not only because they are the main source of dietary calcium, but also because other components may be of interest, such as the lactose content or the bacteria in fermented dairy products. To date, no significant association has been described between calcium intake and colorectal adenomas (Kampman *et al.*, 1994*a*). This finding is all the more important since certain of the current intervention studies with calcium supplements use adenoma recurrence as an end point.

France has a tradition of high calcium intake, mainly in the form of cheese. In order to define further the effect of calcium, phosphate, vitamin D and dairy product intake on the adenoma-carcinoma sequence, we carried out a case-control study in a French community of the relationship between these nutrients, as well as calcium-containing foods and the different macroscopic steps of colorectal carcinogenesis, namely small adenoma, large adenoma and cancer.

### Materials and methods

### *Cases and controls*

A case-control study was set up between 1985 and 1990 to investigate risk factors for the different macroscopic steps of the adenoma-carcinoma sequence. Its general design has already been described (Boutron *et al.*, 1995). It consisted of two parallel case-control studies, one examining risk factors for colorectal adenomas, the other for colorectal cancer. Cases and controls were residents aged 30 to 75 of the Côte d'Or area (Burgundy, France). Exclusion criteria were familial polyposis coli or hereditary non-polyposis colorectal cancer and a previous history of colorectal tumour, inflammatory bowel disease, colectomy or any type of cancer. Sample size calculations were based on fat intake, one of the suspected major risk factors for colorectal cancer. Considering the proportion of the population exposed to a high-fat diet, with a power of 80% to demonstrate a relative risk of 2.0 at the 5% level of significance, it was calculated that at least 140 cases and 280 controls were needed in each group.

Two groups of patients with adenomas and polyp-free controls were selected from the endoscopy lists of all gastroenterologists in the area, whether in private or public practice. Adenomas were subdivided by size: patients with only small adenomas constituted the small adenoma group (85 men and 69 women), patients with at least one adenoma over 10 mm in diameter represented the large adenoma group (129 men and 79 women), whereas subjects without any polyp, either adenomatous or hyperplastic (182 men and 245 women), constituted the polyp-free group. The World Health Organization classification of histological types of polyps (Morson and Sobin, 1976) was used in both laboratories which perform all pathological examinations in the area, and using a consensus about classification achieved in a previous study on adenomas. For the large and small polyp groups and for the polyp-free control group, colonoscopy had to reach at least the sigmoid to descending colon junction and in most cases, when incomplete, was completed by a double contrast barium enema. Colonoscopy reached at least the hepatic flexure in respectively 64.4%, 66.2% and 60.8% of the cases. In the polyp groups, patients with hyperplastic polyps only were excluded from the study.

Cancer cases (109 men and 62 women) were recruited through all specialists in charge of such patients, with the help of the Registry of Digestive Tumours of Burgundy. The control group 'Population controls' (159 men and 150 women), were a random sample of the area in the relevant age group obtained from the census list through the INSEE (National Institute for Statistics and Economical Studies).

The mean age was similar in the two polyp groups ($61.6 \pm 10.2$ for large and $59.5 \pm 11.2$ for small adenomas), but lower in the polyp-free group ($54.1 \pm 14.0$; $P<0.01$). The mean age for cancer cases was $64.2 \pm 10.3$, whereas it was $62.1 \pm 11.6$ in population controls ($P=0.05$). Refusal rates

Correspondence: M-C Boutron, Inserm U290, Hôpital Saint Lazare, 107 rue du Faubourg Saint-Denis, 75010 Paris, France.
Received 19 July 1995; revised 25 January 1996; accepted 25 January 1996

were higher among population controls (46.5%) than in the other groups; 20.1% for the cancer group, 14.0% for the large polyp group, 20.2% for the small polyp group and 22.0% for the polyp-free controls.

### *Data*

The diet history method which was used in this study had been previously validated (Boutron *et al.*, 1989). It was a detailed 2 h questionnaire about the diet in the past year which followed the pattern of meals throughout the day. It was administered at the subjects' homes by a specially trained dietician who also coded the data. A food composition table that used data from available food composition tables and additional information from the food industry had been established for the purpose of the study. It included in particular a detailed list of dairy products. The following broad groups were used: (1) milk (total), subdivided into (2) skim or low-fat milk, (3) full-fat milk, (4) hard and semihard cheese, (5) cottage cheese and (6) yoghurt. The latter is defined in France as a fermented milk containing living *Lactobacillus delbrueckii* subsp. *bulgaricus* and *Streptococcus salivarius* subsp. *thermophilus*. All nutritional data were transformed into a mean daily intake of nutrients. Dietary calcium intake was studied as total calcium, non-dairy calcium, dairy calcium, high-fat dairy calcium (over 20 g fat per g calcium) and low-fat dairy calcium. We also studied dietary vitamin D and phosphorus intakes as well as the calcium–phosphorus ratio.

### *Categories*

For nutrients and widely consumed food items, categories were determined separately by sex, from the distribution into quintiles of each control group, the polyp-free group for the adenoma groups, the population-based controls for the cancer group. For food items which were rarely consumed, two or three groups were established. For full-fat milk, consumers were compared with non-consumers. For cottage cheese and yoghurt, three groups consisted of: no consumption, low and high consumption, the cut-off point between the last two groups being the median intake in consumers. In the male population controls, proportions of non-consumers were 48.4% for cottage cheese, 49.7% for yoghurt and 52.8% for full-fat milk. In the male polyp-free controls, corresponding proportions were 52.2%, 36.8% and 49.5%. In women, non-consumers among population controls were 36.7% for cottage cheese, 29.3% for yoghurt and 46.7% for full-fat milk. Similarly in polyp-free controls, they were 38.4%, 24.1% and 46.1%. Cut-off points for the categories are listed in Table I.

### *Analysis*

In order to follow the general scheme of the adenoma–carcinoma sequence, the small adenoma group was compared with the polyp-free controls, the large adenoma group with the small adenoma group, the cancer group with the population control group. Analyses were performed using comparison of mean daily intakes, after logarithmic transformation for equality of variance and multiple logistic regression controlling for age, sex and caloric intake. Odds ratios for estimating the relative risk for different levels of consumption were calculated using as a reference the category of no or lowest consumption. The statistical significance of each studied variable was tested by the maximum likelihood method.

### Results

### *Calcium, phosphorus, vitamin D and colorectal adenomas*

Mean intake of calcium (±standard deviation) was slightly higher in polyp-free controls compared with small or large adenoma patients in both men and women. In men, it was 1349±533 mg day$^{-1}$ in polyp-free controls, 1221±435 mg day$^{-1}$ in small adenoma patients ($P=0.09$) and 1229±433 mg day$^{-1}$ in large adenoma patients. Values were lower in women, with corresponding figures of 1142±427 mg day$^{-1}$, 1073±385 mg day$^{-1}$ and 1080±422 mg day$^{-1}$. Relative risks of small or large adenomas according to the intake of selected nutrients, controlling for age and caloric intake are presented in Table II. There was no significant effect of calcium, whatever its source, on the risk

**Table I** Cut-off points for the categories of the main studied nutrients and dairy products (quintiles except for yoghurt) among the polyp-free controls and the population controls

| Food item | Sex | Cut-off points 1 | 2 | 3 | 4 |
|---|---|---|---|---|---|
| Polyp-free controls | | | | | |
| Phosphorus (mg day$^{-1}$) | Men | 1241.9 | 1557.4 | 1734.6 | 2038.7 |
| | Women | 1003.4 | 1180.9 | 1337.6 | 1554.3 |
| Calcium (mg day$^{-1}$) | Men | 890.0 | 1166.0 | 1378.6 | 1712.5 |
| | Women | 806.9 | 984.7 | 1156.1 | 1413.0 |
| Vitamin D (μg day$^{-1}$) | Men | 3.0 | 3.9 | 5.0 | 5.3 |
| | Women | 2.4 | 3.2 | 4.0 | 6.4 |
| Milk (g day$^{-1}$) | Men | 14.2 | 60.6 | 149.9 | 270.4 |
| | Women | 18.0 | 46.3 | 98.9 | 230.9 |
| Cheese (g day$^{-1}$) | Men | 34.0 | 52.6 | 71.5 | 95.8 |
| | Women | 25.2 | 43.3 | 62.4 | 85.1 |
| Yoghurt (g day$^{-1}$) | Men | 0 | 62.9 | | |
| | Women | 0 | 71.4 | | |
| Population controls | | | | | |
| Phosphorus (mg day$^{-1}$) | Men | 1127.7 | 1327.5 | 1480.8 | 1743.9 |
| | Women | 900.7 | 1032.7 | 1203.8 | 1393.9 |
| Calcium (mg day$^{-1}$) | Men | 766.4 | 904.8 | 1060.6 | 1287.0 |
| | Women | 722.6 | 873.3 | 1033.9 | 1214.5 |
| Vitamin D (μg day$^{-1}$) | Men | 2.5 | 3.4 | 4.3 | 5.7 |
| | Women | 2.1 | 2.5 | 3.3 | 4.7 |
| Milk (g day$^{-1}$) | Men | 14.2 | 62.7 | 147.9 | 459.4 |
| | Women | 20.0 | 76.3 | 157.8 | 262.6 |
| Cheese (g day$^{-1}$) | Men | 34.0 | 52.6 | 71.5 | 95.8 |
| | Women | 17.6 | 40.0 | 59.4 | 84.3 |
| Yoghurt (g day$^{-1}$) | Men | 0 | 26.3 | | |
| | Women | 0 | 69.8 | | |

of small or large adenomas. Results were similar when considering men and women separately: the odds ratio for the highest quintile of intake compared with the lowest was 0.8 (0.3–2.2) in men and 1.0 (0.4–2.4) in women for small adenomas and 0.8 (0.3–2.4) in men and 1.0 (0.3–3.5) in women for large adenomas. Regarding the type of calcium intake, only non-dairy calcium tended to be associated with a reduced risk of small adenomas in both sexes (OR for the highest quintile 0.6; 0.3–1.1). Regarding large adenomas, there was a suggestion that low-fat calcium might be protective in men (OR highest *vs* lowest quintile, 0.3; 0.1–1.0; $P=0.06$), but not in women (OR, 0.8; 0.2–2.5; $P=0.73$). High dietary intake of phosphorus or a low calcium to phosphorus ratio were not associated with an increased risk of adenomas whatever their size. The highest quintile for that ratio was over 0.9 in men and 1.0 in women, whereas the lowest was below 0.6 in men and 0.7 in women. This yielded a relative risk of 0.9 for both small and large adenomas in men, and 1.3 and 1.4 for small and for large adenomas respectively in women. The highest quintile of phosphorus intake was associated with a reduced risk of small adenomas (OR = 0.5; *P* for trend = 0.06).

A high level of vitamin D intake was inversely related to the risk of small adenomas only in women, with a dose–effect relationship and an odds ratio of 0.4 for the highest level of intake, *P* for trend = 0.04. There was no such association in men where the corresponding figure was 1.2, *P* for trend = 0.78 for intake levels close to those in women. These results were not modified when controlling for calcium intake and interaction factors between calcium and vitamin D intakes were not significant ($P=1.0$ for small and 0.6 for large adenomas).

As some dairy products have a high fat content, we tested whether fat intake acted as a confounder by including fat in the logistic model. This modified neither the direction nor the statistical significance of any of the relative risks calculated with the calorie-adjusted model.

*Calcium, phosphorus, vitamin D and colorectal cancer. (Table III)*

Mean consumptions of calcium were slightly higher in cases than in controls, the difference being on the verge of statistical significance in women after logarithmic transformation of the values. Mean consumptions were $1047 \pm 338$ mg day$^{-1}$ and $1134 \pm 435$ mg day$^{-1}$ in male con-trols and cancer patients respectively. In women, corresponding figures were $1142 \pm 619$ mg day$^{-1}$ and $1000 \pm 368$ mg day$^{-1}$.

There was no significant protective effect of calcium intake on the risk of colorectal cancer, with an odds ratio of 1.9 (0.8–4.7) in men and 1.5 (0.5–4.5) in women for the highest *vs* lowest quintile of intake. Dairy calcium in men and non-dairy calcium in women were even associated with an increased risk of cancer, with figures of 2.7 (1.1–6.3; *P* for trend 0.06) and 2.4 (0.9–6.6; *P* for trend 0.04) respectively for the highest level of intake.

There was no significant association between vitamin D intake and the risk of colorectal cancer and there was no interaction between calcium and vitamin D intakes, *P* for interaction = 0.42. There was a trend toward an increased risk associated with phosphorus intake in women (OR, 3.5; 0.8–15.9; *P* for trend 0.08), but not in men (OR, 1.4; 0.4–4.7). There was no significant interaction between calcium and phosphorus intakes and the highest level of the calcium–phosphorus ratio yielded an odds ratio of 1.3 in both men and women ($P > 0.10$).

**Table II** Relationship between intake of calcium, phosphorous and vitamin D and colorectal adenomas[a], Côte d'Or, 1985–90

| | | | Quintiles 1 | 2 | 3 | 4 | 5 | P-value for trend |
|---|---|---|---|---|---|---|---|---|
| Phosphorus | Small adenomas | *n* case/ctl | 41/85 | 44/86 | 26/85 | 25/86 | 18/85 | |
| | | OR | 1.0 | 1.1 | 0.7 | 0.7 | 0.5 | |
| | | 95% CI | | (0.6–1.9) | (0.4–1.3) | (0.3–1.4) | (0.2–1.2) | 0.06 |
| | Large adenomas | *n* case/ctl | 49/41 | 57/44 | 38/26 | 34/25 | 30/18 | |
| | | OR | 1.0 | 1.1 | 1.2 | 1.2 | 1.4 | |
| | | 95% CI | | (0.6–2.0) | (0.5–2.6) | (0.5–2.7) | (0.5–3.9) | 0.56 |
| Calcium | Small adenomas | *n* case/ctl | 35/85 | 48/86 | 21/85 | 27/86 | 23/85 | |
| | | OR | 1.0 | 1.5 | 0.7 | 1.0 | 0.9 | |
| | | 95% CI | | (0.9–2.6) | (0.4–1.4) | (0.5–1.8) | (0.4–1.7) | 0.28 |
| | Large adenomas | *n* case/ctl | 46/35 | 58/48 | 40/21 | 35/27 | 29/23 | |
| | | OR | 1.0 | 0.9 | 1.3 | 0.9 | 0.9 | |
| | | 95% CI | | (0.5–1.6) | (0.6–2.8) | (0.5–1.9) | (0.4–1.9) | 0.85 |
| Non-dairy calcium | Small adenomas | *n* case/ctl | 46/85 | 35/86 | 28/85 | 23/86 | 22/85 | |
| | | OR | 1.0 | 0.8 | 0.7 | 0.6 | 0.6 | |
| | | 95% CI | | (0.5–1.4) | (0.4–1.2) | (0.3–1.1) | (0.3–1.1) | 0.04 |
| | Large adenomas | *n* case/ctl | 50/46 | 48/35 | 40/28 | 35/23 | 35/22 | |
| | | OR | 1.0 | 1.3 | 1.3 | 1.4 | 1.5 | |
| | | 95% CI | | (0.7–2.3) | (0.7–2.5) | (0.7–2.7) | (0.7–2.9) | 0.26 |
| Dairy calcium | Small adenomas | *n* case/ctl | 28/85 | 43/86 | 35/85 | 25/86 | 23/85 | |
| | | OR | 1.0 | 1.6 | 1.4 | 1.1 | 1.0 | |
| | | 95% CI | | (0.9–2.9) | (0.8–2.5) | (0.6–2.0) | (0.5–2.0) | 0.63 |
| | Large adenomas | *n* case/ctl | 39/28 | 53/43 | 57/35 | 32/25 | 27/23 | |
| | | OR | 1.0 | 0.9 | 1.1 | 0.8 | 0.8 | |
| | | 95% CI | | (0.5–1.6) | (0.6–2.1) | (0.4–1.7) | (0.3–1.7) | 0.59 |
| Vitamin D | Small adenomas | *n* case/ctl | 41/85 | 33/86 | 36/85 | 22/86 | 22/85 | |
| | | OR | 1.0 | 0.9 | 1.0 | 0.6 | 0.7 | |
| | | 95% CI | | (0.5–1.5) | (0.5–1.7) | (0.3–1.2) | 0.4–1.3 | 0.14 |
| | Large adenomas | *n* case/ctl | 49/41 | 49/41 | 48/36 | 34/22 | 28/22 | |
| | | OR | 1.0 | 1.3 | 1.1 | 1.2 | 1.0 | |
| | | 95% CI | | (0.7–2.4) | (0.6–2.0) | (0.6–2.4) | (0.5–2.1) | 0.95 |

[a]Small adenomas compared with polyp-free controls, large compared with small adenomas.

*Dairy products and colorectal cancer (Table III)*

No particular type of dairy product was associated with risk of colorectal cancer, apart from an increased risk in men drinking full-fat milk (OR 1.8; $P=0.03$), which was not observed in women (OR 1.0; $P=0.95$).

*Dairy products and colorectal adenomas (Table IV)*

No particular type of dairy product was significantly associated with the risk of small adenomas. As for large adenoma patients, they consumed significantly less yoghurt than small adenoma patients, with a global odds ratio for men and women combined of 0.6 (0.4–1.1) for consumers of less than half a yoghurt per day, and 0.5 (0.3–1.0) for consumers of at least half a yoghurt per day, compared with subjects who did not consume yoghurt (*P* for trend = 0.03). When controlling for alcohol intake, which has been found to be the only nutrient significantly associated with the risk of large adenomas (Boutron *et al.*, 1995), the association remained but was slightly reduced (OR category 3 *vs* no consumption, 0.6; *P* for trend 0.08). The association was not modified when controlling for fruit intake which was the only other food associated with a reduced risk of large adenomas (Boutron 1995). This inverse association with yoghurt intake was slightly different in men and in women. In men, only the highest level of intake was associated with a reduced risk, whereas in women, the difference was between those who consumed *vs* those who did not consume yoghurt. In addition, consumption of full-fat milk was associated with a moderately increased risk of large adenomas in men (OR 1.7; $P=0.07$) but not in women (OR 0.8; $P=0.45$).

### Discussion

Our results do not support the hypothesis that calcium exerts a protective effect against colorectal tumours. The only exception, and then only in men, was a negative association between low-fat calcium and large adenomas. We even observed a small positive association between calcium intake and risk of colorectal cancer. Vitamin D was inversely related only to the risk of small adenomas in women. Intake of yoghurt was inversely related to the risk of large adenomas.

Retrospective assessment of dietary intake is one limitation of case–control studies. We chose to study the adenoma–carcinoma sequence stepwise in order to reduce the potential impact of dietary changes over time. Changes in diet due to the disease are another concern, but it is unlikely that this bias would explain the failure to observe a protective effect of dietary calcium. Our patients with adenomas had no reason for having modified their diet, because adenomas are mostly asymptomatic, and also because we had excluded subjects with a previous history of adenomas. There is no reason why patients with cancer should have increased their intake of dairy products as a result of their disease. Prospective studies are not submitted to the potential recall bias, and some also failed to observe a protective effect of dietary calcium, and have even found a positive association with cancer (Bostick *et al.*, 1993). The quality of retrospective dietary assessment is another important determinant of the validity of such a study. We performed a pilot study in order to test and adapt available dietary history questionnaires and we validated our questionnaire (Boutron *et al.*, 1989). The way of comparing the different groups of cases and controls is also debatable. In accordance with Hill's hypothesis (Hill *et al.*, 1978) of two

**Table III** Relationship between calcium or dairy products intake and colorectal cancer, Côte d'Or, 1985–90

| | | Quintiles[a] 1 | 2 | 3 | 4 | 5 | *P-value for trend* |
|---|---|---|---|---|---|---|---|
| Phosphorus | *n* case/ctl | 25/61 | 29/62 | 33/62 | 36/62 | 48/62 | |
| | OR | 1.0 | 1.2 | 1.3 | 1.5 | 1.9 | |
| | 95% CI | | (0.6–2.4) | (0.6–2.7) | (0.7–3.3) | (0.8–4.6) | 0.17 |
| Calcium | *n* case/ctl | 25/61 | 34/62 | 34/62 | 27/62 | 51/62 | |
| | OR | 1.0 | 1.3 | 1.3 | 1.0 | 1.7 | |
| | 95% CI | | (0.7–2.5) | (0.7–2.4) | (0.5–1.9) | (0.8–2.3) | 0.33 |
| Non-dairy calcium | *n* case/ctl | 27/61 | 25/62 | 39/62 | 36/62 | 44/62 | |
| | OR | 1.0 | 0.9 | 1.4 | 1.3 | 1.6 | |
| | 95% CI | | (0.5–1.8) | (0.7–2.6) | (0.7–2.5) | (0.8–3.0) | 0.11 |
| Dairy calcium | *n* case/ctl | 22/61 | 36/62 | 31/62 | 35/62 | 47/62 | |
| | OR | 1.0 | 1.5 | 1.3 | 1.5 | 1.8 | |
| | 95% CI | | (0.8–2.9) | (0.7–2.6) | (0.8–2.8) | (0.9–3.4) | 0.17 |
| Vitamin D | *n* case/ctl | 29/61 | 26/62 | 38/62 | 50/62 | 29/62 | |
| | OR | 1.0 | 0.8 | 1.2 | 1.5 | 0.8 | |
| | 95% CI | | (0.4–1.6) | (0.6–2.2) | (0.8–2.9) | (0.4–1.6) | 0.77 |
| Milk (total) | *n* case/ctl | 27/62 | 50/62 | 25/62 | 33/61 | 36/62 | |
| | OR | 1.0 | 1.7 | 0.8 | 1.2 | 1.2 | |
| | 95% CI | | (0.9–3.2) | (0.4–1.6) | (0.6–2.2) | (0.6–2.2) | 0.66 |
| Low fat milk | *n* case/ctl | 29/62 | 27/62 | 49/62 | 35/61 | 31/62 | |
| | OR | 1.0 | 1.0 | 1.6 | 1.2 | 1.0 | |
| | 95% CI | | (0.5–1.8) | (0.9–2.8) | (0.7–2.3) | (0.5–1.9) | 0.76 |
| Cheese | *n* case/ctl | 32/62 | 32/62 | 37/62 | 42/61 | 28/62 | |
| | OR | 1.0 | 1.5 | 1.3 | 1.3 | 1.2 | |
| | 95% CI | | (0.8–2.7) | (0.7–2.4) | (0.7–2.4) | (0.6–2.2) | 0.89 |
| Cottage cheese | *n* case/ctl | 68/132 | 46/87 | 57/90 | | | |
| | OR | 1.0 | 1.1 | 1.2 | | | |
| | 95% CI | | (0.7–1.7) | (0.8–1.9) | | | 0.39 |
| Yoghurt | | 71/123 | 52/93 | 48/93 | | | |
| | | 1.0 | 1.0 | 1.0 | | | |
| | | | (0.7–1.7) | (0.6–1.6) | | | 0.93 |

[a]Quintiles for all foods except yoghurt and cottage cheese (three levels).

steps before cancer itself, i.e. adenoma formation and adenoma growth, we chose to compare our adenoma groups stepwise. It should be emphasised that because the adenoma and polyp-free groups were obtained from endoscopy lists and thus represent a selected group of subjects, odds ratios cannot be assumed to represent population relative risks. They rather provide an estimation of the significance of a risk factor in a selected population. Nevertheless, as adenomas are mostly asymptomatic, and only a small sample of adenoma-bearing subjects are diagnosed, comparing them with our population controls would have introduced bias.

The debate about a possible protective effect of calcium intake on colorectal cancer is relatively recent. The epidemiological evidence relating colorectal cancer and intakes of calcium, vitamin D or dairy products has recently been summarised (Bostick *et al.*, 1993). A protective effect of calcium, not always statistically significant, was suggested in five of nine case–control and in three of five cohort studies (Garland *et al.*, 1985; Stemmermann *et al.*, 1990; Bostick *et al.*, 1993). In a later study in the Netherlands (Kampman *et al.*, 1994*b*), calcium was even positively, although not significantly, associated with cancer risk, as in our own study.

The inconsistency of the effect of calcium on colorectal cancer may be caused by the level of intake in the studied communities. Most studies which observed a significant effect were performed in the USA, where the mean consumption of calcium is lower than in our population (Weaver, 1994). One may hypothesise that only subjects with insufficient calcium intake have a higher risk of colorectal tumours and that doses above a certain level may not be beneficial. In an animal experiment, the effect of bile acids on colonic cell proliferation was enhanced by a calcium-depleted diet (0.1%), but a high daily calcium intake (1%) did not confer any additional protection compared with the standard calcium diet (0.5%) (Piard *et al.*, 1994).

The mechanisms of a possible protective effect of calcium have been mainly investigated through studies of colonic cell proliferation in animals (Piard *et al.*, 1994) and in high-risk subjects (Lipkin and Newmark, 1985). Calcium supplements decreased colonic cell hyperproliferation in subjects with a personal history of colorectal tumours (O'Sullivan *et al.*, 1993). Calcium has been suggested as having a direct effect on colonic cells, inducing terminal differentiation and growth limitation (Buset *et al.*, 1990). Its effect has also been related to the binding of free bile acids (FBA) and fatty acids (FA) to form insoluble soaps (Newmark *et al.*, 1984). Such a reaction depends on the colonic levels of calcium and phosphorus and on the pH. We did not observe any modulating effect of phosphorus on the relationship between calcium and colorectal tumours, possible because in our study population calcium and phosphorus intakes were highly correlated (correlation coefficient above 0.8).

The absence of effect of dietary calcium on risk of adenomas in our study is consistent with all other epidemiological studies on adenomas (Kampman *et al.*, 1994*a*). This contrasts with the protective effect observed on colonic cell proliferation, and raises questions about the mechanisms of action of calcium in the adenoma–carcinoma sequence. If the on-going intervention studies demonstrate a protective effect of calcium supplements on colorectal adenomas, this may lead to advising calcium supplements rather than increasing dietary consumption of calcium.

Three case–control and two cohort studies considered the effect of vitamin D, three suggested a protective effect,

**Table IV** Relationship between dairy products intake and colorectal adenomas[a], Côte d'Or, 1985–90

| | | | Quintiles[b] 1 | 2 | 3 | 4 | 5 | *P-value for trend* |
|---|---|---|---|---|---|---|---|---|
| Milk (total) | Small adenomas | *n* case/ctl | 32/85 | 38/86 | 25/85 | 29/85 | 30/86 | |
| | | OR | 1.0 | 1.3 | 0.8 | 0.9 | 1.1 | |
| | | 95% CI | | (0.7–2.3) | (0.4–1.6) | (0.5–1.7) | (0.6–1.9) | 0.71 |
| | Large adenomas | *n* case/ctl | 41/32 | 44/38 | 34/25 | 49/29 | 40/30 | |
| | | OR | 1.0 | 0.9 | 1.0 | 1.2 | 1.0 | |
| | | 95% CI | | (0.5–1.7) | (0.5–2.0) | (0.6–2.4) | (0.5–2.0) | 0.60 |
| Low-fat milk | Small adenomas | *n* case/ctl | 36/85 | 37/86 | 22/85 | 28/85 | 31/86 | |
| | | OR | 1.0 | 1.2 | 0.7 | 0.8 | 1.0 | |
| | | 95% CI | | (0.7–21) | (0.4–1.3) | (0.5–1.5) | (0.5–1.7) | 0.53 |
| | Large adenomas | *n* case/ctl | 43/36 | 42/37 | 46/22 | 38/28 | 39/31 | |
| | | OR | 1.0 | 0.9 | 1.7 | 1.1 | 1.1 | |
| | | 95% CI | | (0.5–1.8) | (0.9–3.5) | (0.6–2.2) | (0.5–2.1) | 0.64 |
| Cheese | Small adenomas | *n* case/ctl | 29/85 | 50/86 | 20/85 | 26/85 | 29/86 | |
| | | OR | 1.0 | 2.2** | 1.2 | 1.2 | 1.2 | |
| | | 95% CI | | (1.2–3.8) | (0.6–2.2) | (0.6–2.2) | (0.6–2.4) | 0.52 |
| | Large adenomas | *n* case/ctl | 36/29 | 52/50 | 44/20 | 43/26 | 33/29 | |
| | | OR | 1.0 | 0.8 | 1.3 | 1.3 | 1.1 | |
| | | 95% CI | | (0.4–1.6) | (0.7–2.8) | (0.6–2.6) | (0.5–2.5) | 0.33 |
| Cottage cheese | Small adenomas | *n* case/ctl | 65/189 | 42/97 | 47/141 | | | |
| | | OR | 1.0 | 1.3 | 1.0 | | | |
| | | 95% CI | | (0.8–2.1) | (0.7–1.6) | | | 0.79 |
| | Large adenomas | *n* case/ctl | 78/65 | 66/42 | 64/47 | | | |
| | | OR | 1.0 | 1.3 | 1.1 | | | |
| | | 95% CI | | (0.8–2.2) | (0.7–1.8) | | | 0.66 |
| Yoghurt | Small adenomas | *n* case/ctl | 37/126 | 72/148 | 45/153 | | | |
| | | OR | 1.0 | 2.0* | 1.3 | | | |
| | | 95% CI | | (1.3–3.3) | (0.8–2.2) | | | 0.43 |
| | Large adenomas | *n* case/ctl | 75/37 | 89/72 | 44/45 | | | |
| | | OR | 1.0 | 0.6 | 0.5* | | | |
| | | 95% CI | | (0.4–1.1) | (0.3–0.9) | | | 0.03 |

[a]Small adenomas compared with polyp-free controls, large compared with small adenomas. [b]Quintiles for all foods except yoghurt and cottage cheese (three levels). *$P<0.05$; **$P<0.01$.

significant in two (Bostick *et al.*, 1993). We observed an inverse association between vitamin D intake and risk of small adenomas only in women. This may be owing to low consumption levels in our population, as dairy products were not supplemented with vitamin D in France at the time of the study.

Eleven case–control and two cohort studies have investigated the effect of dairy products (Bostick *et al.*, 1993). Nine suggested an inverse relationship which was significant in four. Some dairy products may have a specific effect on colorectal carcinogenesis, independently of their calcium content. It might be suggested that in countries where milk consumption is high, a protective effect of milk itself has been misleadingly attributed to calcium. In lactose malabsorbers, the non-absorbed lactose is fermented in the colon. Fermentation of the closely related lactulose has been demonstrated to be an asset against colorectal carcinogenesis (Nagengast *et al.*, 1988). It decreases intestinal pH, thus reducing the transformation of primary into secondary bile acids. Only populations with a relatively high proportion of lactase-persistent subjects have a tradition of adults drinking large quantities of milk (Sahi, 1994). This might explain in part why dairy products are more often found protective in Anglo-Saxon or Nordic communities than in Latin communities.

Fermented dairy products have been proposed as protective against colorectal carcinogenesis, but the definition of these products differs from one study to another. We decided to study each type separately, as their bacterial content is different. Yoghurt specifically displayed an inverse relationship with risk of large adenomas, suggesting that consumers of at least half a yoghurt per day might be at lesser risk. If this is confirmed, consumption of three yoghurts per week would be easily practicable as a prevention measure. A correlation study in Denmark and Finland suggested a protective role of fermented milk and lactobacilli against colorectal cancer (Report from the IARC Intestinal Microecology Group, 1977), with an incidence rate significantly lower in Finns, who consume larger quantities of fermented dairy products and have a higher rate of lactobacilli in their stools, than Danes. Few epidemiological studies have addressed a possible association between fermented dairy products and colorectal tumours. An inverse association was observed in population-based case–control studies of colon cancer, with yoghurt (Peters *et al.*, 1992) or fermented milk consumption (Young and Wolf, 1988). In contrast, a recent study in the Netherlands (Kampmann *et al.*, 1994*b*) observed no protective effect of fermented dairy products or of yoghurt. A small non-significant inverse relationship between adenomas and fermented dairy products has been observed (Kampmann *et al.*, 1994*a*). The mechanisms by which fermented dairy products might inhibit colon carcinogenesis are speculative. Most of the lactose contained in fermented dairy products is absorbed in the small intestine, even in lactase-deficient subjects (Marteau *et al.*, 1990). Lactic acid bacteria are more likely to be the active component (Rafter, 1995). Some species have been demonstrated to bind carcinogens, decrease the concentration of pro-carcinogen activating enzymes, produce anti-mutagenic compounds or modulate the immune response (Pool-Zobel *et al.*, 1993; Marteau and Rambaud, 1993; Rafter, 1995). However, these properties are strain-specific, and bacterial quantities in experimental studies are usually far higher than those consumed by humans. *L. bulgaricus* and *S. thermophilus* have been less studied with regard to colon carcinogenesis than other bacteria such as *L. acidophilus* or *L. casei.* They are not of human origin and they survive poorly their transit through the stomach and the intestine (Marteau and Rambaud, 1993), but they have exhibited some anti-carcinogenic properties *in vitro* (Pool-Zobel *et al.*, 1993).

In conclusion, this study failed to support a protective effect of dietary calcium on colorectal carcinogenesis, which is in agreement with findings in other Latin countries (Benito *et al.*, 1991). This should prompt further research on the specific effect of certain dairy products. Preventive advice should encourage taking calcium supplements rather than increasing dietary intake of calcium in particular in patients who may otherwise benefit from calcium supplements, such as post-menopausal women for preventing osteoporosis.

**Acknowledgements**
The authors wish to thank Mrs Lieubray, Grillet and Belghiti, who performed the interviews, and Drs Bataillon, Bedenne, Carli, Gambert, Hillon, Jacquot, Klepping, Massart, Meny, Riot, Roy and Villand for their participation. This work was supported by the INSERM (CRE 87-8011) and the Europe against Cancer Program.

### References

BENITO E, STIGGELBOUT A, BOSCH FX, OBRADOR A, KALDOR J, MULET M AND MUNOZ N. (1991). Nutritional factors in colorectal cancer risk: a case–control study in Majorca. *Int. J. Cancer*, **49**, 161–167.

BOSTICK RM, POTTER JD, SELLERS TA, MCKENZIE DR, KUSHI LH AND FOLSOM AR. (1993). Relation of calcium, vitamin D, dairy food intake to incidence of colon cancer among older women. The Iowa women's health study. *Am. J. Epidemiol.*, **137**, 1302–1317.

BOUTRON MC. (1995). Risk factors for colorectal carcinogenesis: epidemiological study following the adenoma–carcinoma sequence. *PhD. thesis*, pp. 169–179. Paris 7 University, Paris.

BOUTRON MC, FAIVRE J, MILAN C, LORCERIE B AND ESTEVE J. (1989). A comparison of two diet history questionnaires that measure usual food intake. *Nutr. Cancer*, **12**, 83–91.

BOUTRON MC, FAIVRE J, DOP MC, QUIPOURT V AND SENESSE P. (1995). Tobacco, alcohol and colectral tumors: a multistep process. *Am. J. Epidermol.*, **141**, 1038–1046.

BUSET M, GALAND P, LIPKIN M, WINAWER S AND FRIEDMAN E. (1990). Injury induced by fatty acids or bile acids in isolated human colonocytes prevented by calcium. *Cancer Lett.*, **50**, 221–226.

GARLAND CF, SHEKELLE RB, BARRETT-CONNOR E, CRIQUI MH, ROSSOF AH AND PAUL O. (1985). Dietary vitamin D and calcium and risk of colorectal cancer: a 19-year prospective study in men. *Lancet*, **1**, 307–309.

HILL MJ, MORSON BC AND BUSSEY HJR. (1978). Aetiology of adenoma–carcinoma sequence in the large bowel. *Lancet*, **1**, 245–247.

KAMPMAN E, GIOVANNUCCI E, VAN'T VEER P, RIMM E, STAMPFER M, COLDITZ GA, KOK FJ AND WILLETT WC. (1994*a*). Calcium, vitamin D, dairy foods, and the occurrence of colorectal adenomas among men and women in two prospective studies. *Am. J. Epidemiol.*, **139**, 16–29.

KAMPMAN E, VAN'T VEER P, HIDDINK GJ, VAN AKEN-SCHNEIJDER P, KOK FJ AND HERMUS RJ. (1994*b*). Fermented dairy products, dietary calcium and colon cancer: a case–control study in the Netherlnds. *Int. J. Cancer*, **59**, 170–176.

LIPKIN M AND NEWMARK H. (1985). Effect of added dietary calcium on colonic epithelial cell proliferation in subjects at high risk for familial colonic cancer. *N. Engl. J. Med.*, **313**, 1381–1384.

MARTEAU P AND RAMBAUD JC. (1993). Potential for using lactic acid bacteria for therapy and immunomodulation in man. *FEMS Microbiol. Rev.*, **12**, 207–220.

MARTEAU P, FLOURIE B, POCHART P, CHASTANG C, DESJEUX JF AND RAMBAUD JC. (1990). Role of the microbial lactase (EC 3.2.123) activity from yoghurt on the intestinal absorption of lactose: an *in vivo* study in lactase-deficient humans. *Br. J. Nutr.*, **64**, 71–79.

MORSON BC AND SOBIN LH. (1976). *Histopathological Typing of Intestinal Tumours.* WHO: Geneva.

NAGENGAST FM, HECTORS MPC, BUYS WAM AND VAN TONGEREN JHM. (1988). Inhibition of secodnary bile acid formation in the large intestine by lactulose in healthy subjects of two different age groups. *Eur. J. Clin. Invest.*, **18**, 56–61.

NEWMARK HL, WARGOVICH MJ AND BRUCE WR. (1984). Colon cancer and dietary fat, phosphate, and calcium: a hypothesis. *J. Natl Cancer Inst.*, **72**, 1323–1325.

O'SULLIVAN KR, MATHIAS PM, BEATTIE S AND O'MORAIN C. (1993). Effect of oral calcium supplementation on colonic crypt cell proliferation in patients with adenomatous polyps of the large bowel. *Eur. J. Gastroenterol. Hepatol.*, **5**, 85–89.

PETERS RK, PIKE MC, GARABRANT D AND MACK TM. (1992). Diet and colon cancer in Los Angeles County, California. *Cancer Causes Control*, **3**, 457–473.

PIARD F, MARTIN M, BOUTRON MC, HILLON P, HAMMAN A AND MARTIN F. (1994). Effect of different doses of dietary calcium on murine colonic cell proliferation. *Eur. J. Cancer Prev.*, **3**, 215–221.

POOL-ZOBEL BL, MUNZNER R AND HOLZAPFEL WH. (1993). Antigenotoxic properties of lactic acid bacteria in the *S. typhimurium* mutagenicity assay. *Nutr. Cancer*, **20**, 261–270.

RAFTER JJ. (1995). The role of lactic acid bacteria in colon cancer prevention. *Scand. J. Gastroenterol.*, **30**, 497–502.

REPORT FROM THE IARC INTESTINAL MICROECOLOGY GROUP. (1977). Dietary fibre, transit time, fecal bacteria, steroids, and colon cancer in two Scandinavian populations. *Lancet*, **2**, 207–211.

SAHI T. (1994). Genetics and epidemiology of adult-type hypolactasia. *Scand. J. Gastroenterol.*, **29 (suppl. 202)**, 7–20.

SLATTERY ML, SORENSON AW AND FORD MH. (1988). Dietary calcium intake as a mitigating factor in colon cancer. *Am. J. Epidemiol.*, **128**, 504–514.

STEMMERMANN GN, NOMURA A AND CHYOU P-H. (1990). The influence of dairy and non-dairy calcium on subsite large-bowel cancer risk. *Dis. Colon Rectum*, **33**, 190–194.

TUYNS AJ, KAAKS R AND HAELTERMAN M. (1988). Colorectal cancer and the consumption of foods: a case–control study in Belgium. *Nutr. Cancer*, **11**, 189–204.

WARGOVICH MJ, ENG VWS, NEWMARK HL AND BRUCE WR. (1983). Calcium amcliorates the toxic effect of deoxycholic acid on colonic epithelium. *Carcinogenesis*, **4**, 1205–1207.

WEAVER CM. (1994). Age-related calcium requirements due to changes in absorption and utilization. *Am. J. Nutr.*, **124**, 1418S–1425S.

WILLETT WC, STAMPFER MJ AND COLDITZ GA. (1990). Relation of meat, fat and fiber intake of colon cancer in a prospective study in women. *N. Engl. J. Med.*, **323**, 1664–1672.

WU AH, PAGANINI-HILL A, ROSS RK AND HENDERSON BF. (1987). Alcohol, physical activity and other risk factors for colorectal cancer: a prospective study. *Br. J. Cancer*, **55**, 687–694.

YOUNG TB AND WOLF DA. (1988). Case–control study of proximal and distal colon cancer and diet in Wisconsin. *Int. J. Cancer*, **42**, 167–175.